# Recent Progress in Research on COVID-19 Pathophysiology: Biomarkers, Repurposed Drugs, Viral Invasiveness, SARS-CoV-2 Genetic Diversity, the Crystal Structure of Viral Proteins, and the Molecular and Cellular Outcomes of COVID-19

**DOI:** 10.3390/ijms232214194

**Published:** 2022-11-17

**Authors:** Jacek Z. Kubiak, Malgorzata Kloc

**Affiliations:** 1 Dynamics and Mechanics of Epithelia Group, Faculty of Medicine, Institute of Genetics and Development of Rennes, University of Rennes, CNRS, UMR 6290, 35000 Rennes, France; 2 Laboratory of Molecular Oncology and Innovative Therapies, Department of Oncology, Military Institute of Medicine, 04-141 Warsaw, Poland; 3 Transplant Immunology, The Houston Methodist Research Institute, Houston, TX 77030, USA; 4 Department of Surgery, The Houston Methodist Hospital, Houston, TX 77030, USA; 5 Department of Genetics, The University of Texas, MD Anderson Cancer Center, Houston, TX 77030, USA

COVID-19 is a disease caused by a novel zoonotic germ known as SARS-CoV-2 coronavirus [1]. The spread of COVID-19 set a precedent in science, because it enabled researchers to follow the emergence of scientific information from point zero, when we knew nothing about the new disease and its cause, to the rapid development of new-generation vaccines and the emergence of new strains of the virus. The flow of information about SARS-CoV-2 coronavirus was initially sluggish. There were more questions than reliable answers. Most speculations about SARS-CoV-2 and COVID-19 came from studies conducted before the pandemic on other coronaviruses transmitted from animals to humans, e.g., SARS in 2002 and MERS in 2012 [2]. However, COVID-19 showed limited similarities to SARS and MERS, making extrapolation of these results to the new disease risky. The common factor was pneumonia, which is caused by all coronaviruses, but the details of the course of these diseases varied widely. We did not know why SARS-CoV-2, unlike SARS and MERS, elicited a variety of often overwhelmingly violent reactions in patients. Also a mystery was the fact that in some patients, SARS-CoV-2 infection is asymptomatic, while others experience only cold-like symptoms, and many others develop severe or even fatal pneumonia. The first scientific reports on the description of the disease and the virus RNA genome sequence [3] came from China, where the pandemic began. One of the first intriguing observations was the report that smokers suffer less from COVID-19 than nonsmokers [4,5,6]. This apparent paradox helped researchers discover that one of the main causes of COVID pneumonia is the so-called cytokine storm, in which an exaggerated reaction of the innate immune system, especially pulmonary macrophages, plays an essential role.

Since the occurrence of these dramatic events in late 2019 and early 2020, enormous progress has been made in research on COVID-19 and SARS-CoV-2. The necessity to overcome the pandemic forced rapid progression of biological, medical, virological, and epidemiological research and its rapid disclosure. Scientific journals published all articles on the new disease in open access mode to accelerate the propagation of knowledge. This process is still ongoing, as evidenced by, among others, the second round of our Special Issue on “Coronavirus Disease (COVID-19): Pathophysiology 2.0”, which we present to our readers.

In this Special Issue of *IJMS*, we present eight original research papers [7,8,9,10,11,12,13,14] and three review articles [15,16,17] addressing several key questions related to molecular aspects of COVID-19 pathophysiology.

Antonio Paolo Beltrami and colleagues [7] analyzed the sera of patients who had “non-severe” and “severe” COVID-19 outcomes. Using machine learning techniques, they identified nine proteins (CD200R1, MCP1, MCP3, IL6, LTBP2, MATN3, TRANCE, α2-MRAP, and KIT) of interest that enabled the correct classification of COVID-19 disease severity in combination with relative neutrophil and lymphocyte counts. They identified nine serum biomarkers corresponding to a “severe” outcome and three others downregulated in the most severe cases. Their article provides potentially very useful predictors of COVID-19 severity in patients.

Hasan Vatandaslar [8] studied the dynamics and efficiency of the reaction of RNA-dependent RNA polymerase (RdRp) inhibition by remdesivir and cordycepin, which are frequently used for SARS-CoV-2 clearance in COVID-19. He focused on the major obstacle to efficient inhibition of this reaction, i.e., competition with endogenous nucleosides. He concluded that the clinically approved concentrations of these drugs for COVID-19 treatment are suboptimal for effective RdRp inhibition. These results should impact the design of new SARS-CoV-2 antiviral compounds.

From the beginning of the COVID-19 pandemic, the repurposing of existing drugs was considered the most rapid way to identify potential new treatments for SARS-CoV-2. Sakshi Piplani and colleagues [9] applied a virtual screening approach to find repurposed drugs against the SARS-CoV-2 helicase (non-structural protein nsp13). Their top hits were not only the expected antivirals, but also antihistamines and antipsychotics. They identified the most promising drug candidates for repurposing, and validated computational predictions using experimental data from previous studies. The authors highlighted that their predictions will require extensive experimental validation.

Anett Hudák and collaborators [10] analyzed the biodistribution and cellular uptake of inactivated SARS-CoV-2 particles in in vitro and in vivo mouse models, taking advantage of the properties of mammalian syndecans. The inactivated SARS-CoV-2 was introduced to several mouse organs, including the brain. The virus raised tissue TNF-α levels, with the highest level in the heart, reflecting inflammatory processes. This study analyzing SARS-CoV-2 cellular entry mechanisms revealed novel aspects of the virus internalization processes and, most importantly, evidenced the ACE2-independent entry routes of SARS-CoV-2. The authors highlighted the potential importance of endocytic pathways in infection with and replication of the virus.

Sara Garcinuño and colleagues [11] studied the process of natural killer cell (NK) degranulation and its correlation with COVID-19 severity in patients. They observed higher NK degranulation and a higher granzyme A/granzyme B ratio in non-severe patients and healthy controls compared to patients with severe COVID-19. They concluded that prompt and efficient NK degranulation during the early stages of infection could enable the identification of patients, leading to a better chance of disease resolution.

Paloma Troyano-Hernáez and co-workers [12] presented a study on the evolution of SARS-CoV-2 in Spain during the first two years of the pandemic. They reported on the circulating SARS-CoV-2 variants in consecutive epidemic waves and analyzed the frequency of mutations, amino acid conservation, and the most frequent amino acid changes in viral proteins. They noticed that viral accessory proteins had more variable positions, while the structural ones had more amino acid changes per sequence. This epidemiological study described six main SARS-CoV-2 lineages, which spread in Spain during the analyzed period between 2020 and 2022.

Chien-Yi Ho et al. [13] conducted a study on the main protease M^pro^, which is an evolutionarily conserved protein among different coronaviruses (CoVs). They focused on canine coronavirus (CCoV) and SARS-CoV-2 and resolved the crystal structure of CCoV M^pro^ in complex with the drug GC376. The structural information presented in this study delivers mechanistic insights into the ligand binding and dimerization of CoV M^pro^s among different species. The species-specific differences in three selected sites of the substrate-binding pocket and two sites of the dimerization interface of M^pro^ seem particularly important in specific monoclonal antibody development and/or antiviral drug design.

When infection begins, SARS-CoV-2 first targets the ciliated cells of the respiratory epithelium. Tom Schreiner and colleagues [14] presented data on the mechanism of ciliary loss and regeneration and discussed the potential long-term consequences of these process for COVID-19.

They worked with an experimental model of SARS-CoV-2 infection in the golden Syrian hamster. Using light transmission microscopy, scanning electron microscopy, and the immunolocalization of activated caspase-3, FOXJ1, p73, and CK14 within the tracheal respiratory epithelium, they followed the loss and regeneration of cilia. The major novelty is that the restoration of ciliation was accompanied by the long-lasting loss of FOXJ11 and an increased frequency of cilia with ultrastructural alterations. This finding indicated that ciliary dyskinesia is related to low FOXJ1 levels.

One of the following three review articles discussed the mechanisms of oxidative stress upon SARS-CoV-2 infection and whether antioxidative drugs can counteract damage caused by the cytokine storm [15]. The second described the role of Nuclear Factor Kappa B (NF-κB) in the development and treatment of COVID-19 [16]. The third discussed the consequences of nicotinamide adenine dinucleotide (NAD+) metabolism dysregulation in SARS-CoV2 infection. These consequences resemble those in pellagra and vitamin B3 deficiency, which cannot be cured by vitamin B3 supplementation [17].

The articles collected in this Special Issue demonstrate enormous progress in understanding COVID-19 pathophysiology, highlight the hottest trends in biomedical research on SARS-CoV-2 infection, and allow us to envision future treatments for the disease.
